# Cardiac tamponade as a rare presentation of Hodgkin’s lymphoma: a case report

**DOI:** 10.3389/fonc.2025.1653256

**Published:** 2025-08-19

**Authors:** Piotr Limanówka, Matylda Kujawińska, Anna Kaput, Aleksandra Spyra, Michał Dobrakowski, Tomasz Szczepański, Aneta Pobudejska-Pieniążek

**Affiliations:** ^1^ Department of Paediatric Haematology and Oncology in Zabrze, Faculty of Medical Sciences in Zabrze, Medical University of Silesia in Katowice, Zabrze, Poland; ^2^ Department of Radiology and Radiodiagnostics, Faculty of Medical Sciences in Zabrze, Medical University of Silesia in Katowice, Zabrze, Poland

**Keywords:** Hodgkin’s lymphoma, cardiac tamponade, radiotherapy, proton beam therapy, case report

## Abstract

Hodgkin’s lymphoma usually manifests with symptoms such as enlarged peripheral lymph nodes, fever, night sweats, and weight loss. It’s the third most common pediatric cancer and includes two main types. Here, we describe a case of a 17-year-old boy, whose one of the first signs of Hodgkin’s lymphoma was cardiac tamponade. Moreover, our patient suffered from another episode of cardiac tamponade after proton beam therapy.

## 1 Introduction

Hodgkin’s lymphoma (HL) is the third most frequent type of pediatric cancer. It’s most common in teenagers, although it can also impact older people. It often presents with enlarged peripheral lymph nodes, night sweats, fatigue, and weight loss (1). The two main types of HL are classical Hodgkin’s lymphoma, which includes nodular sclerosis, mixed-cellularity, lymphocyte-rich, and lymphocyte-depleted subtypes, and nodular lymphocyte-predominant Hodgkin’s lymphoma. The Cotswolds-modified Ann Arbor is a staging system used in HL and includes four stages labelled I, II, III and IV (1). The choice of therapeutic method depends on the stage and begins with chemotherapy. In cases where patients exhibit an unsatisfactory treatment response, radiotherapy is implemented following the completion of all scheduled cycles of chemotherapy (2). Limited research has been conducted on the cardiac implications of HL. Pericardial effusion (PE) is an uncommon symptom in this disease. It is extremely rare for PE to result in cardiac tamponade (CTp) as a sign of HL (3). Here, we report the case of a 17-year-old boy with stage IIB (E-lesions) lymphocyte-depleted classic Hodgkin’s lymphoma (LDHL).

## 2 Case description

**Figure 1:**
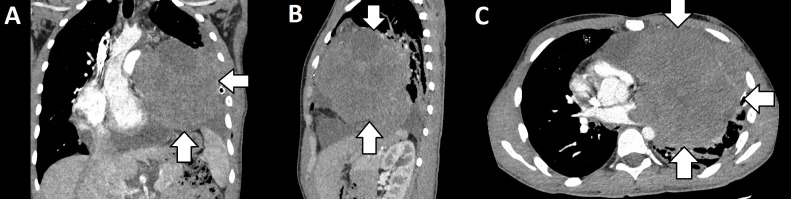
CT images displayed in coronal **(A)**, sagittal **(B)**, and axial **(C)** views showing nodular tissue-cystic mass in the left mediastinum.

In the summer of 2022, the patient experienced an unintentional weight loss of 15 kilograms. Since March of 2023, he has reported night sweats and a decrease in exercise tolerance. The same month he was admitted to the pediatric department. Imaging tests revealed a significant nodular shadow in the projection of the left pulmonary hilum and fluid in the left pleural cavity. CT of the chest showed the presence of a substantial nodular tissue-cystic mass in the left mediastinum. The tumor engorged the pulmonary veins on the left side, displaced the heart, trachea, and mediastinum to the right side, penetrated the aorto-pulmonary window, and extended to the base of the neck. The CT scan obtained at admission is shown in **Figure 1**.

**Figure 2:**
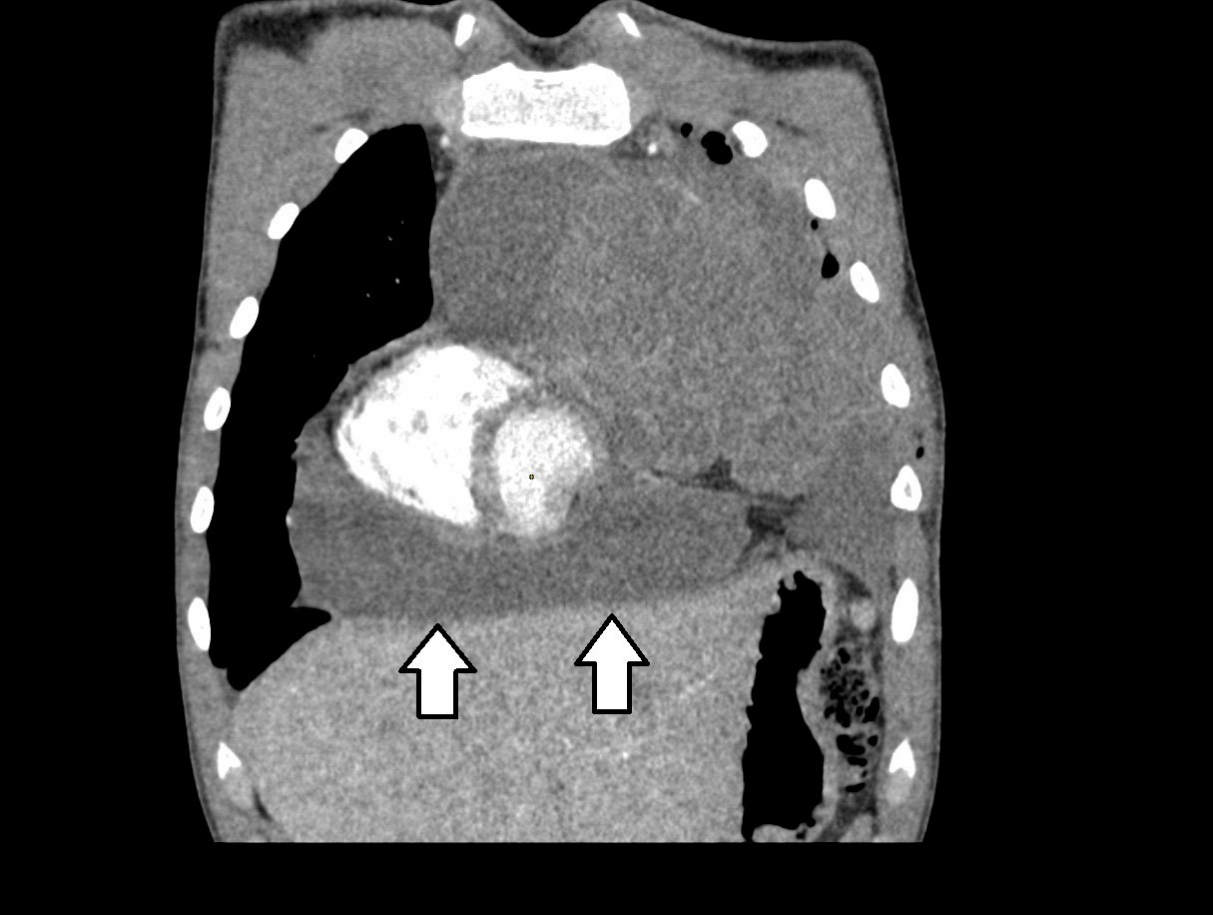
CT image displayed in coronal view, showing fluid within the pericardial sac.

The patient was transferred to the pediatric oncology and hematology department on March 9, 2023, due to a suspected neoplastic process. Peripheral blood count revealed: Hb 10.2g%, CRP 113mg/dl, ESR 43mm/h and coagulation system disruptions: low prothrombin index, high D-dimer level and low AT III level. A sample of the mediastinal tumor was taken for histopathological examination, which confirmed LDHL. Cells with presence of CD30, CD15, PAX-5, MUM.1, and a differential reaction for Bcl-2, with a negative reaction for CD20, CD3 and LMP/EBV were found. On March 20, 2023, a second CT scan was performed with evidence of a 60 mm fluid collection within the pericardial sac (**Figure 2**).

On March 21, 2023, a PET scan was performed to assess the advancement of the disease before starting treatment. During a cardiology consultation the same day, an ECHO examination revealed a significant amount of fluid surrounding the entire heart. Due to the large PE and presence of CTp, it was recommended to transfer the patient to the pediatric cardiology department. On March 22, 2023, the procedure of decompression of CTp was executed, resulting in the extraction of 500 ml of clear fluid. The culture of the fluid, including the detection of tuberculosis infection, was negative, however, cytological examination was not performed at this time. Following the procedure, the patient was readmitted to the pediatric oncology and hematology department. The disease was determined to be stage IIB with E-lesions. The patient was qualified for treatment in accordance with the EURONET-PHL-C2 protocol, therapeutic group TL-3 (4).

Between March 23 and May 4, 2023, two OEPA cycles were carried out. During a follow-up cardiology consultation, a preserved global contractility was described with an ejection fraction of 57%, at the lower limit of the norm. Ramipril 2.5 mg every morning was prescribed. On May 18, 2023, PET scan revealed a metabolically active proliferative process in the nodal mass in the anterior mediastinum with involvement of the left lung (Deauville score 4/5). There was significant but incomplete metabolic and morphological regression compared with the March imaging. Therefore, the patient was qualified for radiotherapy. At the end of May 2023, the first COPDAC-28 cycle was initiated which ended with 4 cycles completed by the end of August.

Preparation for radiotherapy began in September 2023 with a PET/CT scan that showed partial metabolic and morphological regression of the nodal mass compared to the previous examination (Deauville score 4).

From October 4 to October 31, 2023, proton beam radiotherapy was performed with a total dose of 19.8 GyRBE. A subsequent boost was given to the area with a total dose of 29.8 GyRBE. Radiotherapy was complicated by CTp, which was decompressed on October 19. The patient experienced symptoms of CTp, including cough and moderate dyspnea, approximately two weeks after the start of proton therapy. 500 ml of sterile fluid was obtained without cancer cells. The effusion contained 53.13 g/L of protein, while the serum protein level was 63.0 g/L, suggesting an exudative character of fluid. Throughout the entire treatment period, the patient remained under cardiological supervision. Upon examination, a stable, small amount of fluid was detected in the pericardial sac, and subsequently, spironolactone was incorporated into the treatment regimen. At the beginning of February 2024, a PET scan was performed and confirmed metabolic remission. The mass was described as Deauville score 3 with a partial morphological regression.

## 3 Discussion

In 2020, the global age-standardized incidence rate of HL was 0.98 per 100,000 people with the highest incidence rate in the 15–19 age group (5, 6). Among HL survivors, as many as 40% can experience high levels of fatigue or a decline in cognitive performance (1).

Systematic review of lymphoma-associated CTp from 2021 described 52 cases. In the mentioned study 49 patients were diagnosed with non-Hodgkin lymphoma (NHL), which shows that CTp associated with HL can be considered as rare. Additionally, such a phenomenon in children is even more unusual (7). Our patient had subtle shortness of breath in supine position, but was in overall good condition and no cardiac auscultatory changes were found, nor altered blood pressure. Among 52 cases from the mentioned study, only 13 subjects were found to have an increased jugular venous pressure, 12 patients were hypotensive and 13 had distant heart sounds (7). Additionally, Bashir et al. reported that pericardial involvement in lymphoma is usually asymptomatic if it is not substantial (8). However, in our case, even cardiac tamponade presented very limited signs, whereas in other cases, symptoms were more prevalent (3, 9, 10). A possible explanation for generally asymptomatic Ctp in our patient is a long period of development of the disorder, which could result in some degree of adaptation. Nevertheless, it is necessary to remember that the number of cases is very limited.

The patient described in our study had LDHL, which is considered as one of the least common types of HL (1). In other cases of similar patients, types of HL were nodular sclerosing HL (NSHL) (3, 9, 10), which is considered as the most common type (1). Moreover, Bashir et al. reported pericardial involvement in HL as 5% and all of the thirteen subjects had nodular sclerosing tumors. Such a limited number of cases prevents us from describing one type as more susceptible to CTp. However, our patient shows that this such phenomenon is not limited to one type of HL and, to the best of our knowledge, this is the first reported case of CTp in a patient with LDHL.

CTp associated with HL has been described in different situations. The cases mentioned earlier were presented with CTp before any treatment, however it is possible to observe such a disorder in other mechanisms associated with HL. Othman et al. reported a case of a 21-year-old male with HL that presented with CTp, which was supposedly caused by salmonellosis bacteremia (11). Another patient presented progressive pericardial effusion during chemotherapy for HL (12). These two articles show that CTp and pericardial effusion related to HL can be caused by different mechanisms, sometimes unrelated to HL.

Our patient had another episode of cardiac tamponade, but the second time it was caused by proton beam therapy. This way of treating HL is relatively new and has good results. However, it is also related to cardiac toxicity (13). Way et al. described a case of a 25-year-old female with recurrent pericardial effusion presenting during proton beam therapy (14). Similarly, in the case of our patient, cardiac tamponade occurred during this type of therapy. To the best of our knowledge, this case is the first to report CTp associated with proton beam, although it is difficult to indicate the underlying cause of this CTp episode. While current NCCN guidelines do not define a strict cardiological monitoring schedule, our case highlights that cardiac tamponade may occur during proton beam therapy, suggesting that cardiological assessment should be considered during such treatment (15). Although the exact mechanisms are not fully understood, microvascular injury is considered one of the possible causes of pericardial effusion secondary to radiotherapy (16).

## Data Availability

The original contributions presented in the study are included in the article/supplementary material. Further inquiries can be directed to the corresponding authors.
